# Perceptions and emotions of infection control team member during the COVID-19 pandemic in Germany

**DOI:** 10.1017/ash.2022.118

**Published:** 2022-05-16

**Authors:** Sebastian Schulz-Stubner

## Abstract

**Background:** We conducted an anonymous survey in compliance with German data protections regulations among participants of the annual infectious disease and control meeting in Freiburg, Germany, in October 2021. **Methods:** In total, 391 surveys were returned: 188 from nurse infection control practitioners (ICPs) and 66 from specially infection control trained physicians (STPs). We report the results of these 2 subgroups regarding their perceptions and emotions during the pandemic. Descriptive statistics and χ^2^ test with *P* < .05 were used when applicable. **Results:** Shortages of medical masks or FFP2 masks during the first pandemic wave in 2020 were reported by 48.5% STPs and 57.4% ICPs. STPs and ICPs relied equally on information provided by the Robert Koch Institute, the WHO, the ECDC and the CDC. Occupational health information was sought significantly more often by ICPs; only 17% of ICPs never used this source versus 51.5% of STPs (*P* < .001). Most ICPs (58%) and STPs (51%) described their relationship to local authorities as good as well as communication with institutional leaders (69.7%). Fewer ICPs (36.1%) felt frequently appreciated during the pandemic compared to 45.5% of STPs and more ICPs (25%) reported frustration than STPs (18.2%). However, the differences were not statistically significant. Rarely, ICPs (2.1%) or STPs (1.5%) felt unsafe at work and only 1.6% of ICPs and no STPs reported loss of motivation. In addition, 13.8% of ICPs and 12.1% of STPs often felt overwhelmed, but only 3.2% of ICPs and no STPs felt hopeless. Their self-reported competency was rated as high by 75% of ICPs and 69.7% of STPs. The 5 most frequent free-text comments regarding “lessons learned” pertained to better crisis communication, better supply chain management, precise regulations, “less talking more doing,” and mandatory vaccination. The most frequent free-text general comments pertained to maintain basic hygiene measures in private and public life because of the pandemic. **Conclusions:** Our survey results indicate a high level of resilience among members of infections control teams in German medical institutions despite obvious shortcomings in supplies during the first wave of the pandemic. There were no significant differences between physician and nurse members of infection control teams regarding their perceptions and emotions, indicating a homogenous situation within the teams. The high level of self-perceived competency has likely helped deal with the pandemic and prevented the feeling of loss of control implied in the question items “feeling overwhelmed” and “hopeless.”

**Funding:** None

**Disclosures:** None

